# Supplementary material for: Uncovering the characteristics of the gut microbiota in patients with acute ischemic stroke and phlegm-heat syndrome
Source: PLoS One. 2022 Nov 3;17(11):e0276598. doi: 10.1371/journal.pone.0276598 (PMC9632779; doi:10.1371/journal.pone.0276598)
Supplement: S1 Checklist — (PDF) [file pone.0276598.s001.pdf]

STROBE Statement—checklist of items that should be included in reports of observational studies

	Item No.	Recommendation	Page No.	Relevant text from manuscript
**Title and abstract**	1	(*a*) Indicate the study's design with a commonly used term in the title or the abstract	Page 3	This study was designed to investigate the alterations in gut microbiota in patients with AIS and PHS through a cross-sectional study.
		(*b*) Provide in the abstract an informative and balanced summary of what was done and what was found	Page 3	Growing evidence indicates that the characteristics of gut microbiota are associated with acute ischemic stroke (AIS). The long duration of phlegm-heat syndrome (PHS) in patients with AIS could lead to poor clinical outcomes. Gut microbiota characteristics in patients with both AIS and PHS, and their relationship remains unknown.This study was designed to investigate the alterations in gut microbiota in patients with AIS and PHS through a cross-sectional study. Fecal samples were collected from 10 patients with AIS and non-PHS (ntAIS), 7 patients with AIS and PHS (tAIS), and 10 healthy controls (HC). Samples were profiled via Illumina sequencing of the 16S rRNA V3-V4. Stroke severity was assessed at admission by the National Institutes of Health Stroke Scale (NIHSS) and modified Rankin scale (mRS); their correlation with gut microbiota was investigated. The alpha-diversity of the bacterial communities was significantly higher in the fecal samples of patients with tAIS than in patients with ntAIS (Shannon index, P = 0.037). In addition, the combined tAIS and ntAIS group (tntAIS) exhibited higher microbiotic diversity when compared with HC (chao1, P = 0.019). The structure of intestinal microbiota was effectively distinguished between the tAIS and ntAIS group (ANOSIM, r = 0.337, P = 0.007). Additionally, the gut microbiota structure was significantly different between the tntAIS and HC groups (ANOSIM, r = 0.217, P = 0.005). The genera, Ruminococcaceae_ UCG_002 and Christensenellaceae_R-7_group, were implicated in the discrimination of PHS from non-PHS. The

				order Lactobacillales and family Lachnospiraceae were significantly negatively correlated with NIHSS and mRS at admission（P < 0.05). By contrast, the order Desulfovibrionales, families Christensenellaceae and Desulfovibrionaceae, and genera Ruminococcaceae UCG-014 and Ruminococcaceae UCG-002 were significantly positively correlated with NIHSS and mRS at admission（P < 0.05). This study is the first to profile the characteristics of gut microbiota in patients with AIS and PHS, compared with those with non-PHS. The genera, Ruminococcaceae_ UCG_002 and Christensenellaceae_R-7_group, may be objective indicators of this traditional Chinese medicine (TCM) syndrome in AIS. Furthermore, it provides a microbe-inspired biological basis for TCM syndrome differentiation.
**Introduction**				
Background/rationale	2	Explain the scientific background and rationale for the investigation being reported	Page 4	Stroke is the second-leading cause of death and disability worldwide; one out of four individuals will suffer from a stroke in of their lifetime. It is categorized into two types: ischemic and hemorrhagic stroke. In 2009, approximately 62.4% of all stroke events are ischemic. The typical clinical manifestations of AIS is a neurological deficit over a single cerebral arterial vascular territory. At least half of the patients experience gastrointestinal complications after stroke, including intestinal motility dysfunction, intestinal flora disturbance, leaky gut, intestinal bleeding, and enteropathogenic sepsis.<br>Recent studies have indicated that the dysbiosis of gut microbiota plays an essential role in the pathophysiology of neurological diseases, such as Alzheimer’s disease, multiple sclerosis (MS), Parkinson’s disease (PD), and stroke. Additionally, alterations in gut microbiota are a risk factor for stroke; elevated concentrations of opportunistic pathogens and a decreased abundance of butyrate-producing bacteria (BPB) may identify individuals who are at high-

or low-risk for stroke.

Trimethylamine N-oxide (TMAO), a key gut metabolite, which has been shown to play an significant role in the onset, development, and progression of stroke. Interestingly, TMAO has been reported to induce platelet hyperreactivity as a potential mechanism to increase thrombotic risk, suggesting that TMAO increases as a potential risk for acute ischemic events. An animal study demonstrated that the short-chain fatty acid (SCFA) metabolized by gut bacteria could contribute to poststroke neuronal plasticity through promoting microbiota activation and behavioral recovery measured by motor deficits of the affected forelimb, indicating that intestinal microbiota modulation could be a possible therapeutic target for the recovery of stroke. Moreover, studies have confirmed the existence of a bidirectional microbiota–gut–brain axis and the potential of microbiota-based inventions to improve stroke outcomes.

Guided by the theories of Chinese medicine, syndrome differentiation remains at the heart of treatment. A syndrome is commonly known as ‘zheng hou’ in Chinese, which is the awareness of the law of occurrence, development, and manifestation of disease. Syndromes or patterns (“Zheng” in Chinese), defined as symptom clusters, are the specific intervention targets in Chinese medicine theory. Phlegm-heat syndrome (PHS) consists of two syndrome elements, phlegm-damp and heat-flaming. It is the key syndrome in the acute phase of ischemic stroke. Prior studies have indicated that a longer duration of PHS is associated with more severe neurological deficits. In cases of PHS stroke patients, the plasma motilin (MTL) level was obviously increased compared with the other syndrome types, which could further elucidate that stroke patients of PHS were more likely to suffer from the slowing of gastric emptying to release more MTL via neurosecretory feedback.

				Though studies has extensively reported on the relationship between stroke and gut microbiota, little is known about the microbiotic features of the specific TCM syndrome in stroke. In this study, we explored the alterations in gut microbiota in patients with AIS and PHS. Further, we investigated the relationship between gut microbiota and patients severity.
Objectives	3	State specific objectives, including any prespecified hypotheses	Page 5	Though studies has extensively reported on the relationship between stroke and gut microbiota, little is known about the microbiotic features of the specific TCM syndrome in stroke. In this study, we explored the alterations in gut microbiota in patients with AIS and PHS. Further, we investigated the relationship between gut microbiota and patients severity.
**Methods**				
Study design	4	Present key elements of study design early in the paper	Page 3	This study was designed to investigate the alterations in gut microbiota in patients with AIS and PHS through a cross-sectional study.
Setting	5	Describe the setting, locations, and relevant dates, including periods of recruitment, exposure, follow-up, and data collection	Page 6	This study was carried out in the Dongzhimen Hospital, Beijing University of Chinese Medicine. Fecal samples were collected from patients with AIS and non-PHS (ntAIS group, n = 10); AIS and PHS (tAIS group, n = 7); and healthy controls (HC group, n = 10). Recruitment began in November 2021 and continued through the end of January 2022.The first available fresh fecal samples were collected using standard sterile stool collection tubes. Fecal samples from the participants were collected within 48h after the participants were admitted to the hospital.
Participants	6	(*a*) *Cohort study*—Give the eligibility criteria, and the sources and methods of selection of participants. Describe methods of follow-up<br>*Case-control study*—Give the eligibility criteria, and the sources and methods of case ascertainment and control selection. Give the rationale for the choice of cases and controls	Page 6	Eligible subjects were male or female, from the age of 18-85 years. All patients with AIS within seven days of symptom onset were diagnosed according to the guidelines and confirmed by magnetic resonance imaging or computed tomography. The inclusion criterion of the ntAIS group was to have a phlegm-damp and heat-flaming syndrome element score of ≤4. A two syndrome element

		Cross-sectional study—Give the eligibility criteria, and the sources and methods of selection of participants		score of ≥10 was required for inclusion in the tAIS group. These scores were assessed according to Diagnostic Scale of Syndrome Elements in Ischemic Stroke (DSSEIS). Stroke patients with a history of intestinal diseases, intracerebral hemorrhage, other neurological disorders, psychiatric diseases, infectious diseases, autoimmune diseases, malignant tumor or exposure to antibiotics, probiotics, glucocorticoids, or immunosuppressants within 1 month before sample collection were excluded. Patients who were pregnant or reported alcohol abuse were also ineligible for this study. The HC group underwent physical examination; they were absent from parenchymal lesions of the major organs and had no prior history of cerebrovascular disease, including hypertension, diabetes, and hyperlipidemia. HC were screened and excluded if they had taken antibiotics, probiotics, glucocorticoids, or immunosuppressants within one month of the study start date.
		(*b*) *Cohort study*—For matched studies, give matching criteria and number of exposed and unexposed<br>*Case-control study*—For matched studies, give matching criteria and the number of controls per case		
Variables	7	Clearly define all outcomes, exposures, predictors, potential confounders, and effect modifiers. Give diagnostic criteria, if applicable	Page 6	Outcomes:operational taxonomic unit distributions;alpha and beta diversities;Linear discriminant analysis Effect Size (LEfSE) analysis;the relationship between stroke severity and gut microbiota.<br><br>The baseline characteristics of the three groups were consistent and comparable, including age, sex, the body mass index, and comorbidities, excluding the influence of specific comorbidities (hypertension, diabetes, and hyperlipidemia) and other confounders that affect gut microbiota.<br><br>Diagnostic criteria:patients with AIS within seven days of symptom onset were diagnosed according to the guidelines and

				confirmed by magnetic resonance imaging or computed tomography.
Data sources/ measurement	8*	For each variable of interest, give sources of data and details of methods of assessment (measurement). Describe comparability of assessment methods if there is more than one group	Page 7	To exclude the effect of diet on the intestinal microflora, a medical doctor who was also a registered nutritionist in Dongzhimen hospital provided a standardized recipe designed for stroke patients. Diets formulated in this recipe could also meet the daily energy requirements for healthy people. Three meals per day for all the participants in this study were prepared strictly according to the standardized recipe.
Bias	9	Describe any efforts to address potential sources of bias	Page 6,7	The baseline characteristics of the three groups were consistent and comparable, including age, sex, the body mass index, and comorbidities, excluding the influence of specific comorbidities (hypertension, diabetes, and hyperlipidemia) and other confounders that affect gut microbiota.<br><br>To exclude the effect of diet on the intestinal microflora, a medical doctor who was also a registered nutritionist in Dongzhimen hospital provided a standardized recipe designed for stroke patients. Diets formulated in this recipe could also meet the daily energy requirements for healthy people. Three meals per day for all the participants in this study were prepared strictly according to the standardized recipe.
Study size	10	Explain how the study size was arrived at		The sample size for this cross-sectional analysis was calculated based on the summary and investigation of current literature and consideration for the actual situation of operability.

Continued on next page

Quantitative variables	11	Explain how quantitative variables were handled in the analyses. If applicable, describe which groupings were chosen and why	Page 9	Statistical processing was performed using the IBM Statistics SPSS ver.20.0 software.The data are presented as the mean value ± standard deviation (SD). t-tests were performed when data were normally distributed. Chi-square tests were used to examine the general characteristics of the study population. Intergroup differences in the abundance of intestinal flora were explored using the Wilcoxon rank-sum test. The association between intestinal flora and stroke severity at admission was determined using Spearman's rank correlation coefficient. The threshold for statistical significance was set to $p < 0.05$.
Statistical methods	12	(*a*) Describe all statistical methods, including those used to control for confounding	Page 6	Restrictions were utilized for study participants entry conditions to control for confounding.<br>The baseline characteristics of the three groups were consistent and comparable, including age, sex, the body mass index, and comorbidities, excluding the influence of specific comorbidities (hypertension, diabetes, and hyperlipidemia) and other confounders that affect gut microbiota.
		(*b*) Describe any methods used to examine subgroups and interactions		
		(*c*) Explain how missing data were addressed		
		(*d*) *Cohort study*—If applicable, explain how loss to follow-up was addressed<br>*Case-control study*—If applicable, explain how matching of cases and controls was addressed<br>*Cross-sectional study*—If applicable, describe analytical methods taking account of sampling strategy		
		(*e*) Describe any sensitivity analyses		
**Results**				
Participants	13*	(a) Report numbers of individuals at each stage of study—eg numbers potentially eligible, examined for eligibility, confirmed eligible, included in the study, completing follow-up, and analysed	Page 6	Fecal samples were collected from patients with AIS and non-PHS (ntAIS group, n = 10); AIS and PHS (tAIS group, n = 7); and healthy controls (HC group, n = 10).

		(b) Give reasons for non-participation at each stage		
		(c) Consider use of a flow diagram		
Descriptive data	14*	(a) Give characteristics of study participants (eg demographic, clinical, social) and information on exposures and potential confounders	Page 6	The baseline characteristics of the three groups were consistent and comparable, including age, sex, the body mass index, and comorbidities, excluding the influence of specific comorbidities (hypertension, diabetes, and hyperlipidemia) and other confounders that affect gut microbiota (Table 1; all P > 0.05).
		(b) Indicate number of participants with missing data for each variable of interest		
		(c) *Cohort study*—Summarise follow-up time (eg, average and total amount)		
Outcome data	15*	*Cohort study*—Report numbers of outcome events or summary measures over time		
		Case-control study—Report numbers in each exposure category, or summary measures of exposure		
		Cross-sectional study—Report numbers of outcome events or summary measures	Page 3	The alpha-diversity of the bacterial communities was significantly higher in the fecal samples of patients with tAIS than in patients with ntAIS (Shannon index, P = 0.037). In addition, the combined tAIS and ntAIS group (tntAIS) exhibited higher microbiotic diversity when compared with HC (chao1, P = 0.019). The structure of intestinal microbiota was effectively distinguished between the tAIS and ntAIS group (ANOSIM, r = 0.337, P = 0.007). Additionally, the gut microbiota structure was significantly different between the tntAIS and HC groups (ANOSIM, r = 0.217, P = 0.005). The genera, Ruminococcaceae_ UCG_002 and Christensenellaceae_R-7_group, were implicated in the discrimination of PHS from non-PHS. The order Lactobacillales and family Lachnospiraceae were significantly negatively correlated with NIHSS and mRS at admission（P < 0.05). By contrast, the order Desulfovibrionales, families Christensenellaceae and Desulfovibrionaceae, and genera Ruminococcaceae UCG-014

			and Ruminococcaceae UCG-002 were significantly positively correlated with NIHSS and mRS at admission（P < 0.05).
Main results	16	(*a*) Give unadjusted estimates and, if applicable, confounder-adjusted estimates and their precision (eg, 95% confidence interval). Make clear which confounders were adjusted for and why they were included	
		(*b*) Report category boundaries when continuous variables were categorized	
		(*c*) If relevant, consider translating estimates of relative risk into absolute risk for a meaningful time period	

Continued on next page

Other analyses	17	Report other analyses done—eg analyses of subgroups and interactions, and sensitivity analyses		
**Discussion**				
Key results	18	Summarise key results with reference to study objectives	Page 4	The genera, Ruminococcaceae_ UCG_002 and Christensenellaceae_R-7_group, may be objective indicators of this traditional Chinese medicine (TCM) syndrome in AIS.
Limitations	19	Discuss limitations of the study, taking into account sources of potential bias or imprecision. Discuss both direction and magnitude of any potential bias	Page 22	The main limitation of this study is the small sample size. We recruited few fecal tAIS providers to compare with the ntAIS group. This may result in a failure to detect some certain bacteria acting as potential microbial indicators to differentiate this TCM syndrome. Future research into the mechanisms of these gut microbiota in AIS is required.
Interpretation	20	Give a cautious overall interpretation of results considering objectives, limitations, multiplicity of analyses, results from similar studies, and other relevant evidence	Page 18	Interactions between intestinal microbiota and AIS with a TCM syndrome, such as PHS, is an emerging focus for research. This study found alterations in alpha-diversity and structure of gut microbiota in patients with AIS, which were more prevalent in those patients who also showed signs of PHS. Furthermore, the genera, Ruminococcaceae_ UCG_002 and Christensenellaceae_R-7_group, were implicated in the discrimination between PHS and non-PHS in patients with AIS. This could explain, at least in part, why tAIS patients are more likely to suffer from gastrointestinal complications and show more severe neurological deficits. Additionally, we found some gut microbiota were correlated with stroke severity at admission.
Generalisability	21	Discuss the generalisability (external validity) of the study results	Page 22	The main limitation of this study is the small sample size. We recruited few fecal tAIS providers to compare with the ntAIS group. This may result in a failure to detect some certain bacteria acting as potential microbial indicators to

			differentiate this TCM syndrome. Future research into the mechanisms of these gut microbiota in AIS is required.
**Other information**			
Funding	22	Give the source of funding and the role of the funders for the present study and, if applicable, for the original study on which the present article is based	This research was financially supported by National Key R&D program “Evidence-based evaluation and mechanism of Chinese medicine intervention programs in the acute phase of stroke disease [grant number 2018YFC1705000];the Dongzhimen hospital Beijing University of Chinese Medicine Project [grant number DZMKJCX-2020-003].

*Give information separately for cases and controls in case-control studies and, if applicable, for exposed and unexposed groups in cohort and cross-sectional studies.

Note: An Explanation and Elaboration article discusses each checklist item and gives methodological background and published examples of transparent reporting. The STROBE checklist is best used in conjunction with this article (freely available on the Web sites of PLoS Medicine at http://www.plosmedicine.org/, Annals of Internal Medicine at http://www.annals.org/, and Epidemiology at http://www.epidem.com/). Information on the STROBE Initiative is available at www.strobe-statement.org.
