# Supplementary material for: Uncovering the characteristics of the gut microbiota in patients with acute ischemic stroke and phlegm-heat syndrome
Source: PLoS One. 2022 Nov 3;17(11):e0276598. doi: 10.1371/journal.pone.0276598 (PMC9632779; doi:10.1371/journal.pone.0276598)
Supplement: S1 Table — (PDF) [file pone.0276598.s002.pdf]

Table. Standardized recipe designed according to the Chinese Food Guide Pagoda for participants in this study.

	Monday	Tuesday	Wednesday	Thursday	Friday	Saturday	Sunday
Breakfast	✓ milk 400g<br>✓ an egg<br>✓ fried bean sprouts with vinegar sauce 200g	✓ oatmeal 150g<br>✓ an egg<br>✓ chilled Broccoli 100g	✓ soybean milk 400g<br>✓ an egg<br>✓ nuts 30g	✓ coarse cereal porridge 250g<br>✓ an egg<br>✓ chilled Broccoli 100g<br>✓ nuts 30g	✓ milk 400g<br>✓ an egg<br>✓ fried bean sprouts with vinegar sauce 200g	✓ oatmeal 150g<br>✓ an egg<br>✓ chilled Broccoli 100g	✓ soybean milk 400g<br>✓ an egg<br>✓ nuts 30g
Lunch	✓ roasted chicken drumstick 70g<br>✓ sauteed spinach 200g<br>✓ rice 250g<br>✓ orange 100g	✓ fried fish 150g<br>✓ garlic fried cabbage 200g<br>✓ rice 250g<br>✓ apple 200g	✓ mushroom fried with flowering cabbage 250g<br>✓ rice 250g<br>✓ banana 150g	✓ fried beef with soy beans 150g<br>✓ stir-fry agaric with cabbage 200g<br>✓ rice 250g<br>✓ apple 200g	✓ roasted chicken drumstick 70g<br>✓ sauteed spinach 200g<br>✓ rice 250g<br>✓ orange 100g	✓ fried fish 150g<br>✓ garlic Fried cabbage 200g<br>✓ rice 250g<br>✓ apple 200g	✓ mushroom fried with flowering cabbage 250g<br>✓ rice 250g<br>✓ banana 150g
Dinner	✓ cucumber salad 150g<br>✓ fried beef with onion 120g<br>✓ steamed sweet potato 50g<br>✓ orange 100g	✓ colourful pepper salad 150g<br>✓ coarse cereal porridge 100g<br>✓ grilled chicken breast meat 50g	✓ scrambled egg with tomato 200g<br>✓ sweet and sour fillet of pork 150g<br>✓ steamed sweet potato 50g	✓ lettuce salad 150g<br>✓ oatmeal 150g<br>✓ steamed egg custard 50g	✓ cucumber salad 150g<br>✓ fried beef with onion 120g<br>✓ steamed sweet potato 50g<br>✓ orange 100g	✓ colourful pepper salad 150g<br>✓ coarse cereal porridge 100g<br>✓ grilled chicken breast meat 50g	✓ scrambled egg with tomato 200g<br>✓ sweet and sour fillet of pork 150g<br>✓ steamed sweet potato 50g

Chinese Nutrition Society. Chinese dietary guidelines summary (2016) [M] China: People’s Medical Publishing House; 2016.
